# Achievement of Target Blood Pressure Levels among Japanese Workers with Hypertension and Healthy Lifestyle Characteristics Associated with Therapeutic Failure

**DOI:** 10.1371/journal.pone.0133641

**Published:** 2015-07-30

**Authors:** Nagako Kudo, Hirohide Yokokawa, Hiroshi Fukuda, Hironobu Sanada, Yuichi Miwa, Teruhiko Hisaoka, Hiroshi Isonuma

**Affiliations:** 1 Department of General Medicine, Juntendo University School of Medicine, Tokyo, Japan; 2 Tokorozawa Medical Clinic, Tokorozawa City, Saitama, Japan; 3 Division of Health Science Research, Fukushima Welfare Federation of Agricultural Cooperatives, Aizubange Town, Fukushima, Japan; 4 Department of Tumor and Host Bioscience, Fukushima Medical University School of Medicine, Fukushima, Japan; 5 Department of Health Screening, Tokyo Health Service Association, Tokyo, Japan; The University of Manchester, UNITED KINGDOM

## Abstract

**Background:**

Few studies have examined Japanese with regard to the achievement rates for target blood pressure levels, or the relationship between these rates and healthy lifestyle characteristics in patients with hypertension as defined by the newly established hypertension management guidelines (JSH2014). The aim of this study was to elucidate achievement rates and examine healthy lifestyle characteristics associated with achievement status among Japanese.

**Methods:**

This cross-sectional study, conducted in January-December 2012, examined blood pressure control and healthy lifestyle characteristics in 8,001 Japanese workers with hypertension (mean age, 57.0 years; 78.8% were men) who participated in a workplace health checkup. Data were collected from workplace medical checkup records and participants’ self-administered questionnaires. We divided into 5 groups [G1; young, middle-aged, and early-phase elderly patients (65–74 years old) without diabetes mellitus or chronic kidney disease (CKD) (<140/90 mmHg), G2; late-phase elderly patients (≥75 years old) without diabetes mellitus or CKD (<150/90 mmHg), G3; diabetic patients (<130/80 mmHg), G4; patients with CKD (<130/80 mmHg), and G5; patients with cerebrovascular and/or coronary artery diseases (<140/90 mmHg)] according to JSH2014. And then, achievement rates were calculated in each group. Multivariate analysis identified healthy lifestyle characteristics associated with “therapeutic failure” of target blood pressure.

**Results:**

Target blood pressures were achieved by 60.2% of young, middle-aged, and early-phase elderly patients (G1), 71.4% of late-phase elderly patients (G2), 30.5% of diabetic patients (G3), 33.4% of those with chronic kidney disease (G4), and 66.0% of those with cerebrovascular and/or coronary artery diseases (G5). A body mass index of 18.5–24.9 and non-daily alcohol consumption were protective factors, and adequate sleep was found to contribute to therapeutic success.

**Discussion:**

We found low achievement rates for treatment goals among patients with chronic kidney disease and diabetes mellitus. Maintaining an ideal body weight and adequate alcohol consumption may help with blood pressure control. Lifestyle modification may be necessary for better management of hypertension.

## Introduction

Globally, the overall prevalence of higher blood pressure in adults 25 years and older was around 40% in 2008 [1]. This represented a dramatic increase, likely due to population growth and aging, as the number of people with uncontrolled hypertension rose from 600 million in 1980 to nearly 1 billion in 2008 [1]. In Japan, among those over 30 years of age, 60% and 45% of Japanese men and women, respectively, were estimated to have hypertension, to a total of roughly 43 million [2]. Hypertension is well known as a major risk factor for cardiovascular disease and as a major cause of premature death and disability [3]. This health burden is also closely associated with economic burden, not only due to antihypertensive medications but expensive medical costs for percutaneous coronary intervention, surgical treatment, and other procedures which become necessary [4–5]. In addition, hospitalization and treatment often prevent individuals from going to work. In order to promote both primary and secondary prevention, a better system is critically needed for improved management of hypertension.

In 2014, the Japanese Society of Hypertension (JSH) revised the 2009 version of the Japanese Society of Hypertension Guidelines for Management of Hypertension (JSH2009), and published the JSH2014 [2]. Based on accumulated empirical data, the target blood pressure was changed to <140/90 mmHg. In particular, the target level for those with diabetes mellitus or chronic kidney disease (CKD) was established as <130/80 mmHg. Treatment approaches to address this issue emphasize the necessity of lifestyle modification as well as antihypertensive drug therapy. Some studies estimate that 50–60% of hypertension could be prevented by lifestyle modification [6–7], and others purport that lifestyle modifications can lead to a mild decrease in blood pressure, increase the efficacy of antihypertensive drugs, and contribute to the reduction in future medical care costs [2,8–9].

Few studies have examined Japanese workers with regard to the achievement rates for target blood pressure levels, or the relationship between these rates and healthy lifestyle characteristics in patients with hypertension as defined by the newly established hypertension management guidelines (JSH2014). The present study aimed to elucidate achievement rates for target blood pressure levels and examine the healthy lifestyle characteristics associated with achievement status among Japanese workers.

## Materials and Methods

### Study design and participants

**Fig 1:**
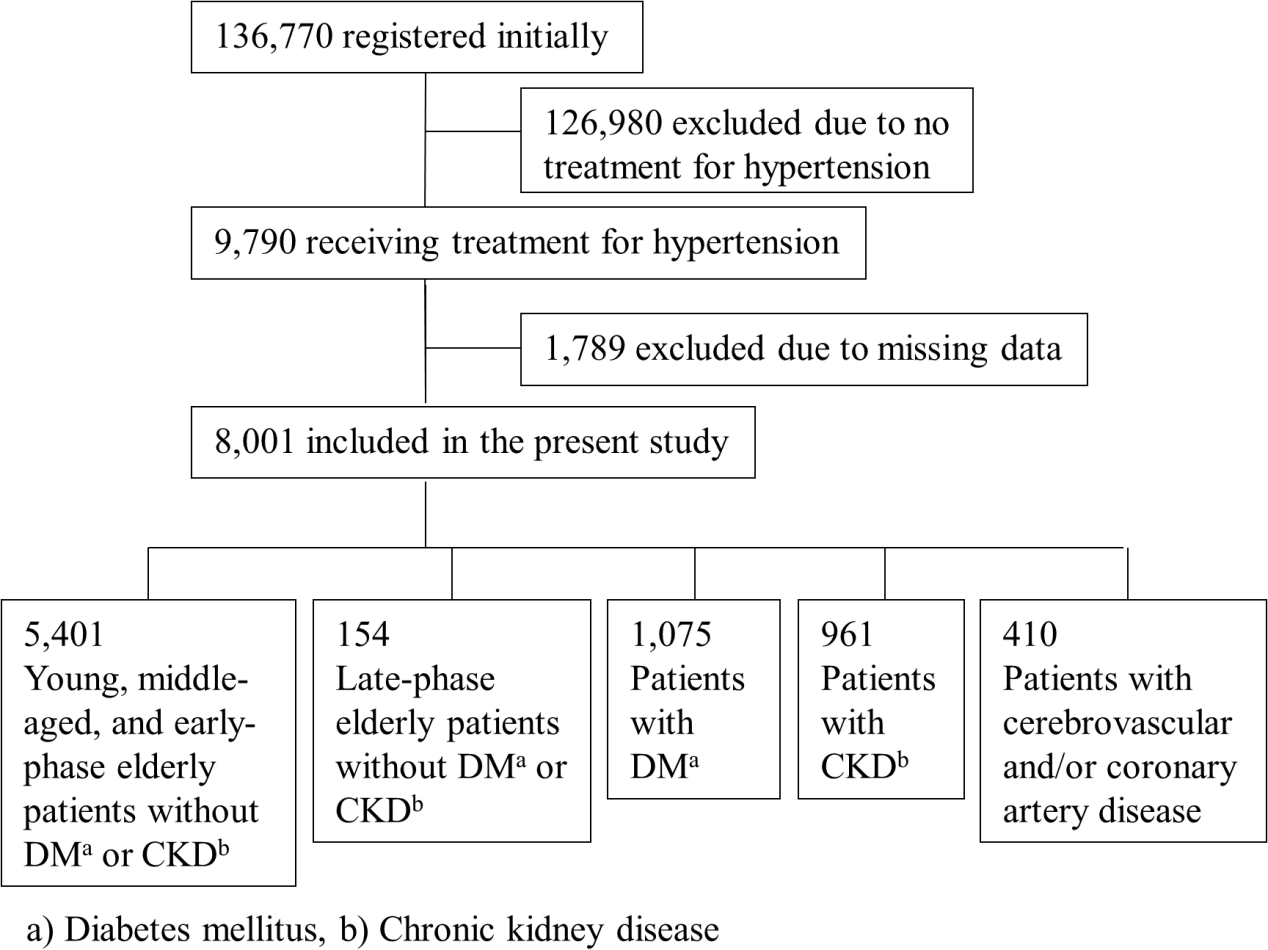
Patients’ registration and flow.

The present cross-sectional study screened 136,770 Japanese individuals who participated in a workplace health checkup conducted by the Tokyo Health Service Association from January to December in 2012 in Tokyo, Japan. Of these, 9,790 who were receiving treatment for hypertension, and 1,789 were excluded due to data missing. Thus 8,001 were included in the present study (Fig 1).

### Study variables

Body height, weight (body mass index; BMI), and waist circumference (WC) were measured in the standing position. BMI was calculated by dividing body weight (kg) by height squared (m^2^). Both systolic blood pressure (SBP) and diastolic blood pressure (DBP) were calculated by calculating the mean of two upper arm blood pressure measurements taken for participants who had been seated for at least five minutes. Serum levels of total cholesterol (mg/dL; TC), high-density lipoprotein cholesterol (mg/dL; HDL-C), low-density lipoprotein cholesterol (mg/dL; LDL-C), and triglycerides (mg/dL; TG) were also measured. LDL-C was estimated using the Friedewald equation [(TC)–(HDL-C)–(TG/5)] [10]. Glycosylated hemoglobin A1c (HbA1c) levels were determined by high-performance liquid chromatography using an automated analyzer. HbA1c [Japanese Diabetes Society (JDS; %)] values were converted to a National Glycohemoglobin Standardization Program (NGSP) equivalent value using the following formula: HbA1c (NGSP) (%) = 1.02 x HbA1c (JDS) (%) + 0.25% [11].

Participants were asked to complete a self-administered questionnaire, which addressed healthy lifestyle characteristics based on Breslow’s seven health practices [12]. These characteristics can be used to assess healthy lifestyle characteristics, and strong associations have been found between healthy lifestyle items and successful blood pressure control among patients with hypertension [13]. Healthy lifestyle items in the questionnaire included non-daily alcohol consumption, non-smoker, exercise frequency of two or more times per week, BMI of 18.5–24.9 kg/m^2^, adequate sleep duration, daily breakfast consumption, and no snacking between meals [12–13].

From the self-administered questionnaire, we also collected information on participants’ present medical history of diabetes mellitus, cerebrovascular diseases, and coronary artery diseases. The following criteria defined CKD: estimated glomerular filtration rate (eGFR) <60 ml/min/1.73 m^2^ and/or urine test of protein ≥2+ on a scale of (–) to (3+) [14].

### Statistical analysis

Results are presented as mean ± standard deviation (SD) for continuous variables or prevalence (%) for categorical variables. Participants were divided into five groups according to categories of JSH2014, and success rates were calculated for each treatment goal as defined by the JSH2014 target values: young, middle-aged, and early-phase elderly patients without diabetes mellitus or CKD (<140/90 mmHg), elderly patients without these diseases (<140/90 mmHg), late-phase elderly patients without diabetes mellitus or CKD (<150/90 mmHg), diabetic patients (<130/80 mmHg), patients with CKD (<130/80 mmHg), and patients with cerebrovascular and/or coronary artery diseases (<140/90 mmHg) [2]. The primary outcome was “therapeutic failure”, defined as not achieving the target blood pressure level. Achievement rates were calculated for each group.

Healthy lifestyle characteristics associated with therapeutic failures were calculated using the odds ratio (OR) and 95% confidence interval (CI) for each item using univariate logistic regression analysis. Following this, factors identified to be significant (P<0.05) by the univariate analysis were entered into the multivariate logistic regression analysis. Late-phase elderly patients without diabetes mellitus or CKD were excluded from the logistic regression analysis, since the number of these participants was relatively small compared to the other groups.

For the two-tailed Student’s t-test, P<0.05 was considered statistically significant. All statistical analyses were performed using the Statistical Package for Social Sciences, version 22 (SPSS Inc, Chicago, IL, USA).

This survey was conducted in compliance with the Ethical Guidelines for Epidemiological Studies established by the Japanese government [15], and in accordance with the Declaration of Helsinki of 1975 (revised in 2000) [16]. The research protocol was reviewed and approved by the Ethics Committee of the Tokyo Health Service Association. The individuals in this manuscript provided written informed consent.

## Results

Mean participant age (SD) was 57.0 (8.8) years, and 78.8% of the participants were men (Table 1). Median BMI was 25.5 (4.0), and median WC was 90.0 (9.6) cm for males and 85.6 (10.3) cm for females. For healthy lifestyle characteristics, 64.0% of participants reported non-daily alcohol consumption, 73.8% were non-smokers, and 84.5% reported no snacking between meals. In addition, 26.3% reported an exercise frequency of 2 times or more per week, and 48.9% had a BMI of 18.5–24.9. Mean SBP and DBP were 133.2 (18.1) mmHg and 82.1 (11.1) mmHg, respectively. The proportion of those with diabetes mellitus was 14.8%, and that with dyslipidemia was 27.0%. Cardiovascular complications were reported in 6.4% of participants, cerebrovascular diseases in 2.1%, low eGFR (<60) in 17.2%, and positive proteinuria in 3.5%.

**Table 1:** Participant characteristics at baseline survey (N = 8,001).

	Mean (SD^a)^) or N (%)	
Age (years)	57.0	(8.8)
Sex (male)	6,306	(78.8)
Anthropometric measurements		
Body mass index	25.5	(4.0)
Waist circumference (cm)		
Men	90.0	(9.6)
Women	85.6	(10.3)
Healthy lifestyle characteristics		
Alcohol consumption (non-daily drinker)	5,122	(64.0)
Smoking behavior (non-smoker)	5,901	(73.8)
Exercise frequency (2 times or more per week)	2,104	(26.3)
Body mass index (18.5–24.9)	3,915	(48.9)
Adequate sleep duration (yes)	4,800	(60.0)
Breakfast (every morning)	6,566	(82.1)
Snack between meals (no)	6,763	(84.5)
Total number of healthy lifestyle characteristics^b)^		
Men	4.5	(1.2)
Women	5.0	(1.2)
Total number of healthy lifestyle characteristics (6–7)	1,776	(22.2)
Hypertension-related factors		
Systolic blood pressure (mmHg)	133.2	(18.1)
Diastolic blood pressure (mmHg)	82.1	(11.1)
Metabolic disorders		
Diabetes mellitus		
Hemoglobin A1c (%)	5.6	(0.9)
Antidiabetic drugs (yes)	1,182	(14.8)
Dyslipidemia		
Total cholesterol (mg/dl)	199.8	(32.6)
High density lipoprotein cholesterol (mg/dl)	56.5	(14.6)
Low density lipoprotein cholesterol (mg/dl)	116.3	(28.2)
Triglycerides (mg/dl)	148.4	(122.9)
Antilipidemic drugs (yes)	2,163	(27.0)
Organ damage/ cardiovascular disease		
Heart	510	(6.4)
Brain	165	(2.1)
Kidney	961	(12.0)
Estimated glomerular filtration rate (ml/min/1.73m^2^)	73.1	(17.2)
Proteinuria (positive)	281	(3.5)

a) Standard deviation

b) Total number of healthy lifestyle characteristics was calculated by summing the items listed in Breslow’s seven health practices associated with mortality.

Table 2 displays mean blood pressures and achievement rates for target blood pressure levels. Young, middle-aged, and early-phase elderly patients without diabetes mellitus or CKD showed mean SBP and DBP of 133.2 (17.7) and 83.0 (10.8) mmHg, respectively. These same measurements were 138.2 (20.8) and 77.2 (10.3) mmHg for late-phase elderly patients without diabetes mellitus or CKD, 134.8 (18.0) and 80.6 (10.9) mmHg for diabetic patients, 132.2 (19.6) and 80.3 (12.3) mmHg for those with CKD, and 130.0 (18.6) and 80.2 (11.3) mmHg for those with cerebrovascular and/or coronary artery diseases. Achievement rates for treatment goals (as defined by JSH2014) were 60.2%, 71.4%, 30.5%, 33.4%, and 66.0%, respectively.

**Table 2:** Mean blood pressures and achievement rates for target blood pressure levels.

	JSH2014 Target blood pressure level (mmHg)	Mean (SD^a)^) systolic and diastolic blood pressures (mmHg)	Achievement rates [N (%)]	
Young, middle-aged, and early-phase elderly patients without diabetes mellitus or chronic kidney disease (n = 5,401)	<140/90	133.2 (17.7) / 83.0 (10.8)	3250	(60.2)
Late-phase elderly patients without diabetes mellitus or chronic kidney disease (n = 154)	<150/90	138.2 (20.8) / 77.2 (10.3)	110	(71.4)
Diabetic patients (n = 1,074)	<130/80	134.8 (18.0) / 80.6 (10.9)	328	(30.5)
Patients with chronic kidney disease (n = 961)	<130/80	132.2 (19.6) / 80.3 (12.3)	321	(33.4)
Patients with cerebrovascular and/or coronary artery disease (n = 410)	<140/90	130.0 (18.6) / 80.2 (11.3)	270	(66.0)

a) Standard deviation.

Multivariate analysis revealed that the factors significantly associated with failure to achieve treatment goals were adequate sleep duration (OR = 1.21, 95%CI = 1.06–1.36), and a BMI of 18.5–24.9 (OR = 0.77, 95%CI = 0.69–0.87) for young, middle-aged, and early-phase elderly patients without diabetes mellitus or CKD (Table 3). For diabetic patients, significant factors were non-daily alcohol consumption (OR = 0.62, 95%CI = 0.48–0.81) and a BMI of 18.5–24.9 (OR = 0.65, 95%CI = 0.50–0.85) (Table 4). For patients with CKD, a BMI of 18.5–24.9 (OR = 0.69, 95%CI = 0.53–0.90) was a significant factor associated with achievement rates (Table 5), while non-daily alcohol consumption (OR = 0.57, 95%CI = 0.37–0.88) showed a significant association in patients with cerebrovascular and/or coronary artery diseases (Table 6).

**Table 3:** Factors associated with therapeutic failure in young, middle-aged, and early-phase elderly patients without diabetes mellitus or chronic kidney disease (n = 5397) (logistic regression analysis).

	Univariate			Multivariate		
	OR^a)^	95% CI^b)^	*P*	OR^a)^	95% CI^b)^	*P*
Adequate sleep duration (yes)	1.20	1.10–1.35	**	1.21	1.08–1.36	**
Body mass index (18.5–24.9)	0.79	0.71–0.89	**	0.78	0.69–0.87	**

a) Odds ratio

b) 95% confidence interval

*P<0.05

**P<0.01.

**Table 4:** Factors associated with therapeutic failure in failure in diabetes mellitus (n = 1,074) (logistic regression analysis).

	Univariate			Multivariate		
	OR^a)^	95% CI^b)^	*P*	OR^a)^	95% CI^b)^	*P*
Alcohol consumption (non-daily drinker)	0.71	0.53–0.95	*	0.62	0.48–0.81	**
Body mass index (18.5–24.9)	0.66	0.50–0.87	**	0.65	0.50–0.85	**

a) Odds ratio

b) 95% confidence interval

*P<0.05

**P<0.01.

**Table 5:** Factors associated with therapeutic failure in chronic kidney disease (n = 961) (logistic regression analysis).

	Univariate			Multivariate		
	OR^a)^	95% CI^b)^	*P*	OR^a)^	95% CI^b)^	*P*
Body mass index (18.5–24.9)	0.69	0.53–0.90	**	-	-	

a) Odds ratio

b) 95% confidence interval

*P<0.05

**P<0.01.

**Table 6:** Factors associated with therapeutic failure in patients with cerebrovascular and/or coronary artery disease (n = 409) (logistic regression analysis).

	Univariate			Multivariate		
	OR^a)^	95% CI^b)^	*P*	OR^a)^	95% CI^b)^	*P*
Adequate sleep duration (yes)	1.58	1.01–2.45	*	1.45	0.93–2.28	
Alcohol consumption (non-daily drinker)	0.50	0.33–0.76	**	0.57	0.37–0.88	**

a) Odds ratio

b) 95% confidence interval

*P<0.05

**P<0.01.

## Discussion

The present study examined data from a large number of participants who underwent a workplace health checkup, revealing achievement rates of target blood pressure levels as defined by the newly published Japanese hypertension treatment guidelines. Among our participants, achievement rates were especially low in patients with diabetes mellitus and CKD. In addition, we found that adequate alcohol consumption and maintaining an ideal body weight may improve blood pressure management.

We found low achievement rates of target blood pressure levels in those with diabetes mellitus and CKD. The JSH2014 emphasizes that the target blood pressure level for hypertensive patients with diabetes mellitus and/or CKD should be less than 130/80 mmHg, because the incidences of cerebrovascular disease and coronary artery disease increase markedly when these diseases are concomitantly present [2]. The different achievement rates observed in the present study could be due to several reasons. First, patients with diabetes mellitus or CKD often have difficulties controlling their blood pressure to the strict target levels. The Chronic Renal Insufficiency Cohort (CRIC) Study, a cross-sectional study of 3,612 participants with CKD in the United States, reported a hypertension prevalence of 85.7%; 98.9% of the participants were aware of this diagnosis and 98.3% were treated with medication, but only 67.1% and 46.1% had controlled their blood pressure levels to <140/90 and <130/80 mm Hg, respectively [17]. A cross-sectional nationally representative health examination survey (NHANES III) examined 9,803 hypertensive participants aged 60 or older and found that a history of diabetes mellitus or CKD was significantly associated with less hypertension control [18]. Also, the Japan Home Versus Office Blood Pressure Measurement Evaluation (J-HOME) study examined 466 Japanese individuals with hypertension and diabetes mellitus and reported that home blood pressure was properly controlled in 18% of participants when the home blood pressure threshold was set tentatively at 130/80 mmHg; the same trend was observed for office blood pressure levels [19]. These findings may indicate the necessity to focus more on these patients, and may highlight the importance of aggressive management of target blood pressure levels to contribute to primary and secondary prevention.

The second potential explanation is a lower awareness of hypertension guidelines in primary care. A study conducted in the United States queried 1,200 primary care physicians via questionnaire, and reported their findings that many physicians have higher blood pressure thresholds for the diagnosis and treatment of hypertension than the 140/90 mmHg criterion recommended by the JNC. This particular study concluded that further improvements in population hypertension control will require behavioral changes among physicians [20]. The Reassessing European Attitudes about Cardiovascular Treatment (REACT) survey, which surveyed 754 randomly selected primary care physicians in five European countries (France, Germany, Italy, Sweden, and the UK), reported that 89% of them were in agreement with the content of current guidelines and 81% reported use of them. However, only 18% of physicians believed that guidelines were being implemented widely [21]. Although the majority of physicians support the concept of guidelines, recommendations may vary by the degree of acceptance. Therefore, it is necessary for general physicians to improve awareness regarding treatment goals recommended by the guidelines and consider the risk factors for each individual. Final explanation is different thresholds among subgroups. Overall results seemed to show excellent control of blood pressure with mean blood pressures <140/90 mmHg among all subgroups. However, patients with DM and/or CKD are required lower target blood pressure goal as <130/80mmHg by JSH 2014. In the United States, the panel members appointed to the Eighth Joint National Committee (JNC 8; 2014 BP guideline) proposed less restrictive BP targets for adults aged 60 years or older and for those with diabetes and chronic kidney disease compared to JSH2014 [22]. A large scale cross-sectional study estimate the proportion of US adults potentially affected by recent changes in recommendations for management of hypertension, and reported that among patients with treatment-eligible hypertension, 40.6% had achieved goal BP under JNC 7, which increased to 56.5% under the 2014 BP guideline [23]. In spite of different thresholds, adequate blood pressure control based on hypertension management guideline is required.

In the present study, maintaining an ideal BMI and non-excessive alcohol consumption helped to prevent failure to achieve treatment goals. Our data showed that half of our participants were obese and only 29.0% exercised at a frequency of 2 or more times per week. Obesity is a classic risk factor for elevated blood pressure and is also well known as an important risk factor for several lifestyle-related disorders [24–27]. A study from Taiwan that examined 89,857 Taiwanese adult patients with type 2 diabetes mellitus reported that the adjusted OR for hypertension for every 1 kg/m^2^ increment of BMI was 1.16 (1.15–1.17) and 1.13 (1.12–1.14) for men and women, respectively [25]. In addition, a cross-sectional analysis of adults enrolled in the Third National Health and Nutrition Examination Survey (NHANES III) found that the OR for hypertension for every 5-unit increase in BMI was 1.45, and that the magnitude of the relative increase in the OR for hypertension was higher among younger adults [26]. The Nord-Trondelag Health Study (HUNT) was conducted as a prospective population study targeting 5,971 individuals in Norway, and found that increases in BMI and decreases in BMI were significantly correlated with increases in SBP and decreases in DBP, respectively, relative to a stable BMI. This trend was observed in both genders and across all age groups [27]. Thus, maintaining an ideal body weight is necessary in order to maintain better blood pressure control. Better hypertension management may also be achieved through non-excessive alcohol consumption and maintaining an ideal body weight [28–30]. One Japanese prospective cohort study observed 3,900 participants for seven years and reported that the annual increase in SBP was 0.44 mmHg greater in those consuming 300 g the amount of ethanol consumption /week or more, relative to non-drinkers, after adjusting for age and weight changes [28]. A large-scale worldwide survey showed that after accounting for key confounders, on average, relative to non-drinkers, men who drank 300–499 ml alcohol/week had systolic/diastolic blood pressures of 2.7/1.6 mmHg higher, while men who drank 500 ml alcohol/week or more had pressures of 4.6/3.0 mmHg higher. Among women, heavy drinkers (300 ml/week or more) also had blood pressures that were 3.9/3.1 mmHg higher than those of non-drinkers [29]. Consistent with this, a meta-analysis found that alcohol restriction has a hypotensive effect [30]. Our data suggested that several healthy lifestyle characteristics may contribute to better blood pressure control and that an assessment of patient lifestyle characteristics and modification of these characteristics in actual clinical settings may be necessary.

One somewhat contradictory finding of the present study was the observation that adequate sleep duration was a risk factor for therapeutic failure among young, middle-aged, and early-phase elderly patients without diabetes mellitus or CKD. Several previous reports found an association between sleep quality and blood pressure controls [31–32]. The Finn-Home study found that self-reported sleep disorders are associated with greater variability in home blood pressure and heart rate measurements [31]. However, another study reported that neither sleep duration nor sleep quality affected the prevalence of hypertension among non-insomniac elderly subjects [32]. One limitation of the present study was that we could not adjust for sleep duration as listed in Breslow’s seven health practices or obtain details on sleep quality using a specific questionnaire. Future studies should examine this issue using detailed data adjusted for sleep-related variables.

Our study has several limitations worth noting. First, selection bias may have been present, as participants comprised only those who underwent workplace medical examinations in Tokyo, Japan. As such, these participants may be inherently more aware of health behaviors relative to residents in rural areas. Further analyses which include data from a more diverse cohort are necessary. Second, some key data were not collected, such as information on medications for hypertension. Third, data collection was conducted in 2012. While the draft of the JSH2014 was introduced in 2013, the JSH2014 was established in 2014. Despite the lag, however, the threshold for patients with diabetes mellitus and chronic kidney disease was very similar to that of the JSH2009, so our results may still be applicable. Further studies after 2014 will be needed. Forth, some lifestyle criteria are vague because lifestyle related items were collected from existing questionnaires. Further studies using a questionnaire focused on lifestyle characteristics will be needed. Fifth, the JSH 2014 guideline differs from the majority of guidelines published elsewhere in the world, such as the JNC 8 guideline 2014 (<140/90 mmHg), American and the International Societies of Hypertension guidelines 2014 (<140/90 mmHg), and the 2013 ESH/ESC (the European Society of Hypertension/the European Society of Cardiology) guideline for the management of arterial hypertension (<140/85 mmHg). Thus, it may be required to pay attention applying our results in other countries. Finally, our study employed a cross-sectional analysis to examine lifestyle characteristics, and thus causal relationships between achievement rates and healthy lifestyles cannot be confirmed. Further analyses of follow-up survey data are needed.

## Conclusion

The present study revealed low achievement rates for treatment goals among hypertensive patients, especially for patients with CKD and diabetes mellitus. With regard to healthy lifestyle characteristics, maintaining an ideal body weight and non-excessive alcohol consumption may contribute to better control of blood pressure levels. Our data indicate the need to focus on these patients and provide instruction regarding lifestyle modification to allow for better management of hypertension according to treatment guidelines.

## Supporting Information

S1 File Basic data base. Data are available from the Tokyo Health Service Association Institutional Data Access / Ethics Committee for researchers who meet the criteria for access to confidential data. The data are under the ethical restrictions of the Ethics Committee of the Tokyo Health Service Association (koho@yobouigaku-tokyo.jp).
(XLSX)
